# ‘Born before arrival’: user and provider perspectives on health facility childbirths in Kapiri Mposhi district, Zambia

**DOI:** 10.1186/1471-2393-14-323

**Published:** 2014-09-16

**Authors:** Selia Ng’anjo Phiri, Knut Fylkesnes, Ana Lorena Ruano, Karen Marie Moland

**Affiliations:** Centre for International Health, Department of Global Health and Primary Care, University of Bergen, Bergen, Norway

**Keywords:** Home deliveries, Health facility childbirth, Born before arrival, Responsiveness, Zambia

## Abstract

**Background:**

Maternal mortality remains high in sub-Saharan Africa. Health facility intra-partum strategies with skilled birth attendance have been shown to be most effective to address maternal mortality. In Zambia, the health policy for pregnant women is to have facility childbirth, but less than half of the women utilize the facilities for delivery. ‘Born before arrival’ (BBA) describes childbirth that occurs outside health facility. With the aim to increase our understanding of trust in facility birth care we explored how users and providers perceived the low utilization of health facilities during childbirth.

**Methods:**

A qualitative study was conducted in Kapiri Mposhi, Zambia. Focus group discussions with antenatal clinic and outpatient department attendees were conducted in 2008 as part of the Response to Accountable priority setting and Trust in health systems project, (REACT). In-depth interviews conducted with women who delivered at home, their husbands, community leaders, traditional birth attendants, and midwives were added in 2011. Information was collected on perceptions and experiences of home and health facility childbirth, and reasons for not utilizing a facility at delivery. Data were analysed by inductive content analysis.

**Results:**

Perspectives of users and providers were grouped under themes that included experiences related to promotion of facility childbirth, responsiveness of health care providers, and giving birth at home. Trust and quality of care were important when individuals seek facility childbirth. Safety, privacy and confidentiality encouraged facility childbirth. Poor attitudes of health providers, long distances and lack of transport to facilities, costs to buy delivery kits, and cultural ideals that local herbs speed up labour and women should exhibit endurance at childbirth discouraged facility childbirth.

**Conclusion:**

Trust and perceived quality of care were important and influenced health care seeking at childbirth. Interventions that include both the demand and supply sides of services with prioritizing needs of the community could substantially improve trust and utilization of facilities at childbirth, and accelerate efforts to achieve MDG5.

**Electronic supplementary material:**

The online version of this article (doi:10.1186/1471-2393-14-323) contains supplementary material, which is available to authorized users.

## Background

The global burden of maternal mortality remains high with 287,000 women reported to have died from pregnancy related complications in 2010 [1]. Maternal mortality is highest in sub-Saharan Africa and south-east Asia, which together account for 85% of the global burden [1]. To reduce this burden the Millennium Development Goal five (MDG 5) aims to achieve a 75% reduction in maternal mortality ratio from 1990 to 2015. The progress has been slow in sub-Saharan Africa [2] despite campaigns like Campaign for Accelerated Reduction of Maternal Mortality in Africa (CARMMA), which aims to promote maternal, new-born and child health. In Zambia the national health strategic plan targets to reduce maternal mortality ratio from the current 591 per 100,000 to 159 per 100,000 live births by 2015 [3].

Many women continue to give birth outside health facilities without skilled attendance with the risk of severe complications and mortality. The proportion of women having facility childbirth, which closely corresponds to delivery with skilled assistance, has not improved much over the years, with reported coverage of 47% in 1996, 44% in 2001–2002 [4, 5] and 48% in 2010 [6]. The government has set out to increase facility deliveries from 28% in 2007 to 50% in 2015 for rural areas and from 79% to 90% for urban areas [3].

Health facility intra-partum strategies, with a skilled birth attendant and efficient referral of pregnancy and childbirth complications, have been shown to be the most effective to address maternal and early neonatal mortality [7, 8]. Skilled birth attendance, which comprises a skilled attendant and an environment providing emergency obstetric care services, is important because complications at delivery may occur without recognizable risk factors during the antenatal period, and response to complications need to be timely [9]. A skilled birth attendant is ‘an accredited health professional – such as a midwife, doctor or nurse – who has been trained to proficiency in the skills needed to manage normal (uncomplicated) pregnancies, childbirth and the immediate postnatal period, and in the identification, management and referral of complications in women and new-borns’ [10].

‘Born before arrival’ refers to childbirth which occurs without a skilled birth attendant and that occurs either at home or en route to a delivery centre or hospital [11]. It is assumed that such deliveries happen elsewhere due to delays in seeking or accessing health facility care. The Zambian health policy encourages all pregnant women to seek skilled birth attendance through facility childbirths in order to have timely management of complications at birth.

To increase the use of skilled birth attendance it is important that interventions target both the demand and the supply of services [12]. The demand side involves the need to utilize healthcare services by individuals, household or the community [13], whereas the supply side involves provision of services by the healthcare system. The health system’s intrinsic goals are to improve the health of the population, to enhance the responsiveness to legitimate expectations of the population, and to create fair financing and financial risk protection [14]. Responsiveness to expectations of the population comprises respect of individuals and client-oriented services that lead to user satisfaction. Fair financing of the health system requires that households are not impoverished, or pay an excessive amount of their income in accessing healthcare. Community perception of whether these goals are met is likely to affect trust, acceptability and utilization of healthcare.

Patient trust is a multidimensional construct that has been approached in medical research in different ways: as a set of beliefs or expectations that a care provider will behave in a certain way, as confidence and reliance in the provider and in the provider’s intent, competence, compassion, privacy and confidentiality, as well as reliability and dependability, and communication [15]. Hence, patient trust is likely to benefit maternal and new-born health by motivating utilization of facilities if the health system adopted a patient-centred care.

This study was part of a multi-centre research project conducted in one district in Kenya, Tanzania and Zambia, the “Response to Accountable priority setting for Trust in health systems” (REACT), which aimed to strengthen the legitimacy and fairness of priority setting at district level with focus on equity, quality and trust in health systems [16]. Our previous study based on data from a cross-sectional survey conducted in the three districts revealed that facility childbirth was associated with striking socio-economic and geographic inequities but it did not seem to be associated with trust and perceived quality of care in the Kenyan and Zambian districts [17]. In the subsequent study we further investigated the low utilisation of health facility birth care in the district in Zambia focusing on trust. Through a qualitative study design we explored perceptions and experiences of users and providers about health care seeking at childbirth.

## Methods

### Setting

The study was conducted in Kapiri Mposhi, a predominantly rural district in the Central Province of Zambia. The district has a population of about 240,000, and with a total fertility rate of 6.4 [6]. The main economic activity is subsistence agriculture. Kapiri Mposhi has one district hospital, which opened in 2011 and provides comprehensive emergency obstetric and neonatal care (EmONC). There are 20 public health centres and two mission health centres. Facility childbirth services are provided almost entirely by the public sector, though the majority of women in the rural area deliver at home assisted by traditional birth attendants or relatives [17]. The health centres operate several outreach clinics to remote areas where they provide maternal and child health services, mainly antenatal, family planning, under-five clinics and rolled out PMTCT. There are also mothers’ shelters where women from remote villages wait before onset of labour. Free ambulance service is provided for patients who have to be transferred from health centres to the hospital for comprehensive EmONC.

### Sample selection and data collection

**Figure 1:**
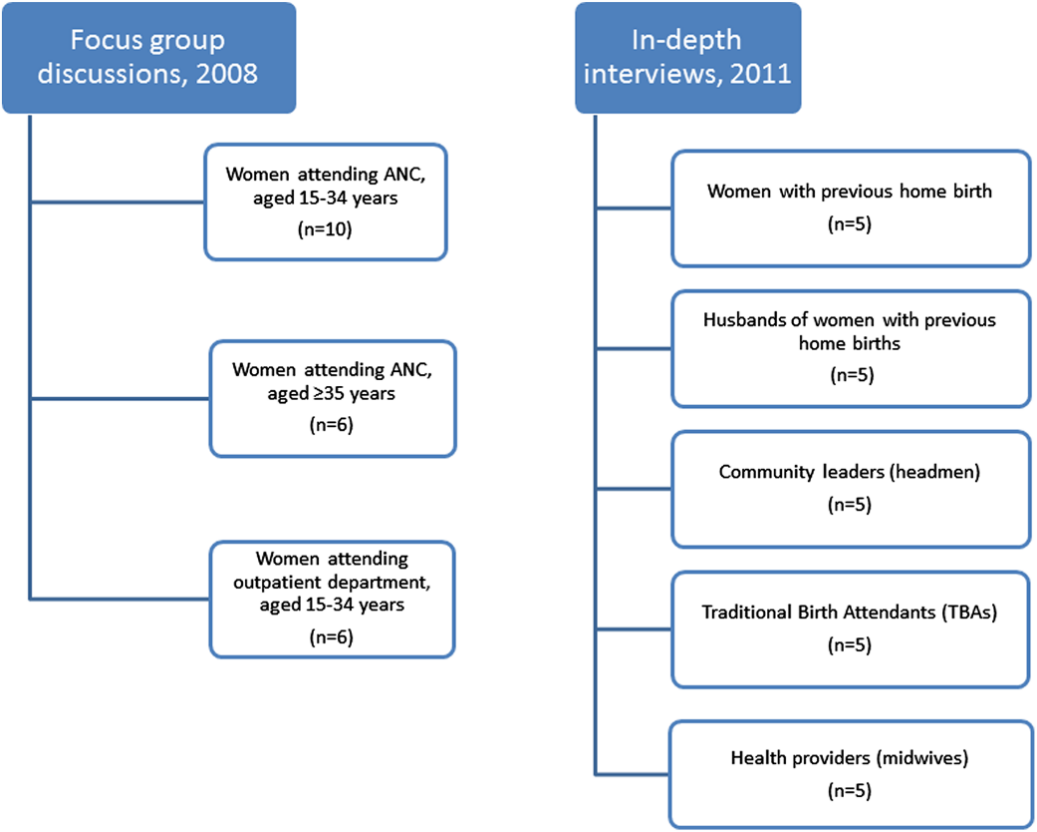
**Composition of participants in focus group discussions and in-depth interviews in Kapiri Mposhi, Zambia.**

Qualitative methods were chosen in order to explore how individuals’ and communities’ perceptions and experiences influence utilization of health care services. Data were collected in two phases. The first phase involved focus group discussions, conducted in July 2008, as part of the REACT project [16]. We conducted FGDs with women attending the antenatal clinic (ANC) and outpatient services at the hospital (Figure 1). Participants were selected by convenient sampling and invited without prior announcement. A topic guide with focus on trust was used and the FGDs, which were facilitated by experienced social science researchers and a research assistant with teaching background, were conducted at the health facilities (see Additional file 1). The topic guide included themes on how trust in the health system was viewed by participants. Questions included perceptions and experiences about what was perceived as the best place to give birth, the most common place to give birth and reasons behind choices of birthing place; about availability of drugs and the conduct of health providers in general and during childbirth in particular; and how health providers could modify their conduct to improve the community’s confidence in them. The topic guide was pre-tested and later validated during the process of conducting the discussions by adding and deleting questions as new themes emerged to obtain rich descriptions but maintained the focus of the guide. Two thirds of women in the FGDs had either attained only primary school education or no education (Table 1). Some had previous home deliveries while others had delivered from a health facility. The discussions were conducted in the local language, tape-recorded and transcribed by the facilitators.

**Table 1:** **Characteristics of women participants in the focus group discussions in Kapiri Mposhi, Zambia in 2008**

Characteristic	OPD* women	ANC ^†^women (15–34 years)	ANC women (≥35 years)
	Number (n = 6)	Number (n = 10)	Number (n = 6)
**Education**			
No education	0	0	2
Primary education (Grade 1–7)	4	5	4
Junior secondary education (Grade 8–9)	2	0	0
Senior secondary education (Grade 10–12)	0	5	0
**Age group (years)**			
15 - 19	1	4	-
20 - 24	2	2	-
25 - 29	1	4	-
30 - 34	2	-	4
35 - 39	0	-	2
40 - 44	0	-	0
**Marital status**			
Single	0	0	0
Married	6	10	6
**Parity**			
0	0	5	0
1 - 3	4	4	3
≥ 4	2	1	3
**Length of stay in Kapiri Mposhi (years)**			
3 - 5	0	5	1
5 - 10	2	3	1
11 - 20	1	1	1
21 - 30	2	1	2
≥ 31	1	0	1
**Occupation**			
Housewife	3	5	0
Marketeer	1	0	4
Self-employed/small scale business	0	2	2
Subsistence farming	2	1	0
Tailor	0	1	0
Student	0	1	0
**Walking distance from home to health** **facility (minutes)**			
<30	0	2	2
30 - 60	6	8	3
>60	0	0	1

*OPD – Outpatient department.

^†^ANC – Antenatal care.

In the second phase in 2011, in-depth interviews (IDIs) were conducted with 25 local stakeholders, as follow up of a quantitative study in the same area. Participants from five selected health centres and their catchment areas were interviewed using semi-structured interview guides. Purposive sampling was employed to recruit the study participants in order to obtain broader perspectives of views and ensure adequate representation of the local community. One community leader was selected from within each catchment area of the health centre. Women and their husbands, and government trained TBAs eligible for the individual interviews were identified by community leaders. Inclusion criteria for the women and their husbands were that they came from the catchment areas of the health centres, and had previous home birth, and had lived continuously for three or more years in the area. The TBAs who were interviewed had lived for three years or more in the area and had facilitated in deliveries within a home environment. Midwives from selected health centres who had served in the area for more than three years were eligible, and were interviewed in order to get the public healthcare provider perspective. Interviews included information about socio-demographic characteristics, perceptions about risks in pregnancy, behaviour relating to pregnancy, utilisation of health facilities during pregnancy and childbirth, and factors that led to home deliveries (see Additional file 2). Validation of the interview guide was done during the process of data collection by clarifying any questions to respondents whenever needed and discussing the first few interviews amongst the researchers before completion of all the interviews. Some interviews were conducted in the villages, whereas others were conducted at health facilities, all by the first author and two assistants. The first author, a Zambian obstetrician practicing in a different part of the country, could understand the local language but not speak it fluently and so interviewed midwives in English. The two research assistants, a teacher and a nurse midwife both fluent with the local language, interviewed the rest of the participants. Rapport with the participants was created and confidentiality assured in order for them to feel confident enough to engage in discussion. All interviews were tape-recorded. Each interview took from 45 minutes to one hour. Transcribing was done verbatim by one research assistant and counter checked by the first author by listening to the tape-recorded interviews.

### Data analysis

**Figure 2:**
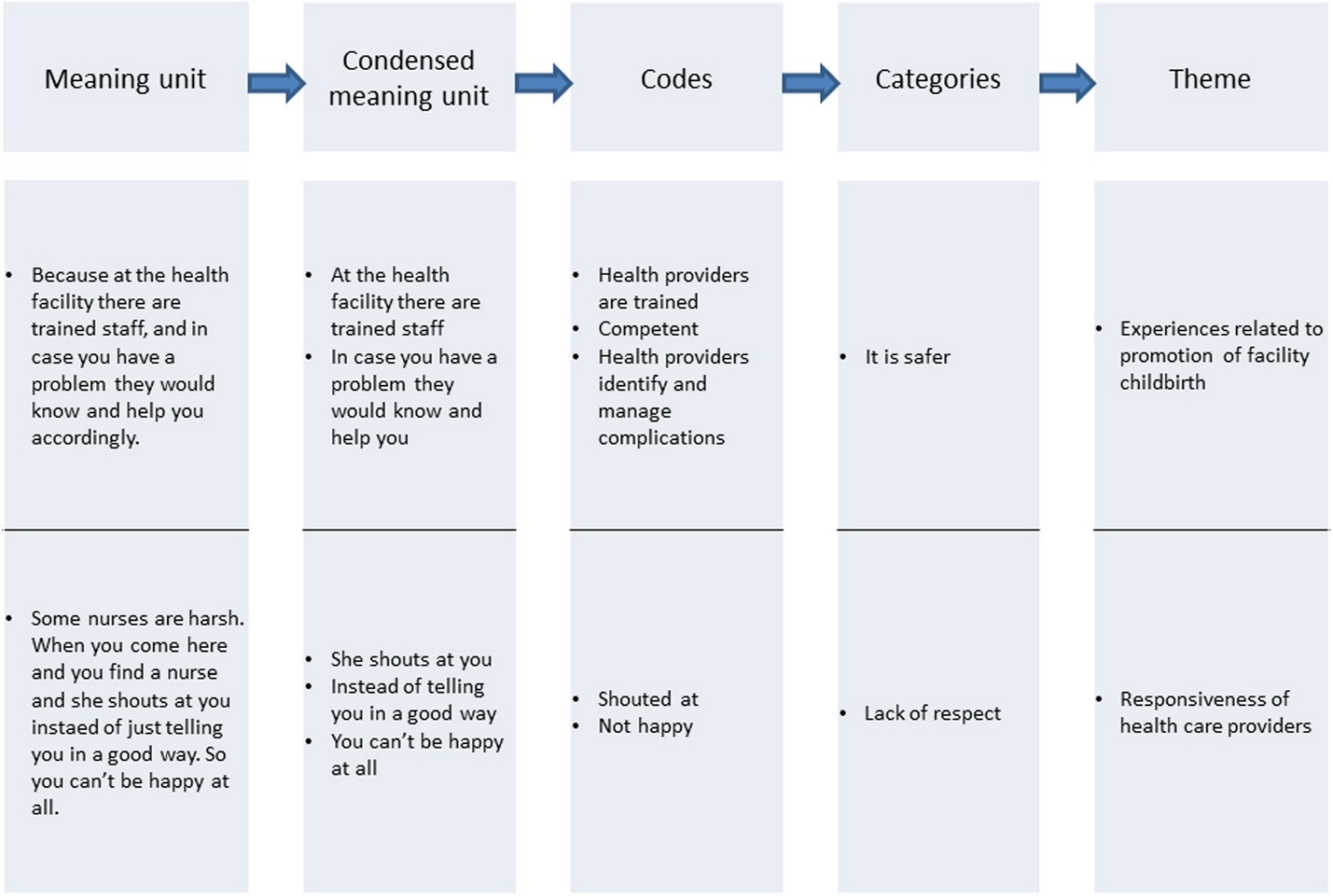
**Example of meaning unit, condensed meaning unit, codes, categories and themes from interviewed participants in Kapiri Mposhi, Zambia.**

The analysis was done stepwise by drawing upon qualitative content analysis [18]. The first step of the analysis was to read through the transcripts holistically and several times in order to get an overview of findings. The second step was to code individual interviews into meaning units, the third was to condense codes into categories based on recurrent patterns and as a fourth step we identified the themes (Figure 2). We used the concepts of responsiveness and trust to interpret the findings. NVivo 10 software was used to organize the data. To validate the coding process we used two independent coders for two of the transcripts and compared the code lists. The categories and themes were discussed among all authors. This research adhered to the BMC guidelines for qualitative research review (RATS).

### Ethical approval

Ethical clearance was obtained from the University of Zambia Ethics Committee. Permission was also granted from Kapiri Mposhi district health management office. Written informed consent was obtained from all participants prior to the interview. Confidentiality and anonymity of study participants was maintained.

## Results

Perspectives of users and providers were grouped under main themes as experiences related to the promotion of facility childbirth, responsiveness of health care providers, and giving birth at home.

### Experiences related to the promotion of facility births

#### Pressure to give birth in health facility

As part of the campaigns to promote facility births the government sensitized communities on the importance of facility childbirth for all women to ensure safety of both mothers and new-born babies. Nurses in local health centres, TBAs and village headmen acted as extensions for the government at the community level and assisted in the sensitization process. Nurses expressed that they gave health education during antenatal care on the importance of utilizing health facilities for safe childbirth while community educational messages were given primarily by TBAs. The TBAs explained that the health policy discouraged them from conducting deliveries in homes except where a woman had progressed in labour and had inadequate time to reach the nearest health facility. The TBAs’ role was redefined from birth attendance to escorting women in labour to the nearest health centre. They explained that the high prevalence of HIV also led to the government advising TBAs to avoid conducting home deliveries. Considering this policy, childbirth that occurred outside the health facility, either at home or en route to the facility, was reported as ‘born before arrival’. As a community leader put it:

*“It is because it is like a law that anyone pregnant should deliver at a health centre…..”* (Community leader, Kapiri Mposhi)

#### It is safer

Health facility delivery was perceived by users, both with previous home and facility childbirths, as safe for both pregnant women and their babies. The qualification of health workers seemed to be an important aspect of health facility birth ‘being safer’. Prevention of Mother to Child Transmission (PMTCT) of HIV services, the presence of trained personnel to identify and manage complications during childbirth and the provision of referral services were all appreciated by users. TBAs described pregnancy as a dangerous period and some described it as “death” because of the complications they encountered. Health providers also noted that excessive bleeding due to retained placenta was a major cause of mortality for women who delivered outside health facilities. The biggest fear that women and men had was the risk of excessive bleeding at delivery.

*“There are some people who bleed a lot during labour, so they find help. And also the baby’s cord is cut in a good way here, and they put a cord clamp which holds well, it doesn’t bleed. But if you give birth at home, most of the time the person helping you to deliver is not even trained and you can end up with a problem”.* (Woman, St. Pauls).

Availability of drugs and equipment was perceived as another advantage for facility childbirth that promoted a more comfortable and safe delivery. Women appreciated receiving pills or injections in a health facility for pain relief. Equipment to monitor blood pressure during labour was seen as an asset of health facility delivery and promoted a feeling of safety.

#### Freedom from gossip and social control

Maintaining privacy and confidentiality by health providers were valued and perceived by women and their husbands to be an advantage of health facilities over the home environment. At home many people were present during the delivery and went around discussing their experience. Women felt it was shameful to have their experiences during labour discussed by others outside the delivery environment. Community leaders, TBAs and midwives agreed that health facilities offered more privacy than at home since only the midwife and one close female relative to the woman would be present in the delivery room. This privacy gave women a sense of safe space to express feelings of pain freely:

*“…..even if you scream in pain, no one will go around telling people about you, but at home some people talk a lot and they go round telling others about you and your experiences”.* (Woman, Kapiri Mposhi).

Despite positive perceptions of health facility childbirth, other experiences such as the responsiveness of health care providers, the fear of giving birth before arriving at a health facility and values associated with enduring pain discouraged the community from using facilities at birth.

### Responsiveness of health care providers

The attitude of health providers such as lack of respect, not providing adequate information and lack of prompt attention discouraged health facility birth. Some health providers gave no information about clinical procedures before performing them on women. This lack of information led to fear of complications and generated anxiety among women, especially the ones that experienced pregnancy for the first time:

*“You come with the first pregnancy and you do not know what to do, they are just saying open your legs, you don’t even know how to give birth, that way a child can even die, you just find that the baby has drowned (aspirated) because you do not know what to do…. they do not even show you what to do”.* (FGD, ANC Women 15-34 yrs)

Experiences differed with some women reporting being welcomed, while others complained of negative attitudes from health providers. The complaints included being shouted at for not pushing well enough during childbirth and being ridiculed for childbearing after age forty. Women perceived this as disrespectful behaviour and experienced as shameful. Other women experienced severe delays in receiving clinical attention within the delivery room and felt neglected and sometimes exposed to risk:

*“Me at one time I was in labour. Instead of examining me, the nurse was just chatting until she knocked off around 18:00 hrs. That nurse did not examine me but luckily the other sister who was reporting (in the shift that followed) came fast. She asked if I had already been examined and I said no. So when she tried to examine me, the baby was already coming out…..and I delivered. If that Mrs M. didn’t come early, I don’t know what would have happened. Up to this time I don’t want to see the other (first) sister in my sight…. I would have died that day”.* (FGD, OPD Women 15-34 yrs)

Poor outcome of their new-born babies was perceived as negligence and having a still-birth in a health facility was a very traumatic experience that could challenge the perception of health facility as the safest place to give birth. However, as the quote below illustrates, a caring nurse may change this perception:

*“…… Like in my case I lost a child and I was not happy with them. But the next one that followed I was well cared for by the nurse on duty on that day. Even now, I still thank her, if I had money I would give her all the time. She looked after me like a small child. She rubbed me and gave me an injection. But for the one I lost I was not happy”.* (FGD, ANC Women, 35 yrs and over)

### Giving birth at home

#### Enduring the pain of labour

There were two dimensions to understanding why women delayed informing their husbands when labour pain started. Prolonged labour in this community was perceived as a sign of infidelity by the husband or wife and so the women seemed reluctant to seek help or inform the husbands early. The connection of prolonged labour and adultery was clearly brought out when a husband was asked how maternal deaths could be prevented and he responded that ‘*women should stop sleeping around*’. This sentiment can be summed up in the local concept of *inchila*, which indicates a prolonged and complicated labour that results in delivery by caesarean section or even death due to infidelity.

Another dimension of delay in reporting labour was to show strength. Bearing the pain with strength and endurance during labour was highly valued for many women. When in labour, the women informed their husbands first, who in turn made arrangements for transport or called for the TBA or a female relative to assist. Husbands and TBAs felt that women tried to show braveness and strength by allowing labour to progress before informing them. Arriving at the health facility in early labour and waiting many hours before giving birth would be considered as being ‘weak’. ‘It was too quick’ was a common way in which most women expressed the reason for having delivered at home. The women mentioned that they had prepared for facility childbirth by buying the delivery kit and had attended ANC, but labour had progressed fast and resulted in home delivery. One woman whose labour normally took six hours, and walking to the nearest health centre normally took about three hours said: *“…what happened was that it was too quick. I even got prepared to start coming to the health centre but I couldn’t walk and I just delivered from home.”* (Woman, Mukonchi). Another woman said her labour usually took on average eight hours, but the last which was a twin delivery took three hours. Walking distance to the nearest health centre was two hours, and her husband said: *“…… but what led her to deliver from home is because it was an emergency (meaning quick progress of labour). We had a plan for her to deliver from the health centre”.* (Husband, Nkole)

Women usually drank traditional herbs in order to avoid prolonged labour and prevent delivery by caesarian section. Herbal medicines were believed to widen the pelvis and accelerate labour pains. The commonly used medicine called *palibe kantu* literally means ‘there is nothing’ indicating that the obstacle to deliver has been removed. The use of herbal medicines was not allowed by health care providers because of the likelihood to result in precipitate labour, complications to the unborn baby and rupture of the uterus. TBAs believed that strong contractions of labour and quick progress stimulated by herbs made it difficult for women to walk and reach the health facility before delivery.

*“….. Because what happens is that as you are escorting her to the clinic, you find she delivers on the way. Then when you ask her what she took, she says her grandmother gave her something to drink so that she can quickly deliver”.* (TBA, Kapiri Mposhi)

#### Delivering before arrival

The cost of preparing for facility childbirth also contributed to delay in seeking facility childbirth. Women had to buy antiseptics and gloves due to shortage of supplies in health facilities. They also needed to buy new baby clothes in preparation for childbirth. When they did not buy these items due to lack of money, women feared the embarrassment of being shouted at by midwives or ridiculed by other women. Most of the women and their husbands in the remote areas had financial constraints since they relied economically on subsistence farming. When weighing possibilities of a complication occurring at childbirth and the costs to purchase a delivery kit combined with transport, many decided to ‘take chances’ and risk having childbirth at home. Although midwives said they attended to women even when they did not have the required items, TBAs and women perceived the need to buy delivery kits as a barrier for facility birth.

*“…. sometimes it is because we don’t manage to buy what we are asked to buy at the facility. Things like jik, dish, chitenge (a ladies wrapper), bucket, new nappies and others, so you decide to die at home. You take a chance.….. And if you go without these items, you are scared to be shouted at….”* (Woman, Kapiri Mposhi)

Women also feared the possibility of delivering on the way to the health facility because of long distances. Most times women in labour were escorted and had to walk or use either ox-carts or bicycles. Bicycle ambulances were introduced to remote areas to facilitate transportation of women in labour, but they were kept at health centres which were located far from the community.

*“Though we have got this “Zamup” ambulance (bicycle ambulance), somebody is in labour and stays very far, maybe 25 kilometers away. The husband comes here, he collects the ambulance, and by the time he reaches the village, maybe he will find she has already delivered. So, long distances”.* (Midwife)

Delivery on the way to the facility was feared because of the bodily exposure even to men and lack of privacy which would be particularly shameful:

*“And sometimes if they use a bicycle, maybe labour progresses and she then delivers on the way, and you know it’s the men who carry us on the bicycles, so it doesn’t look okay if that happens. So, some prefer to deliver at home for fear that they might deliver on the way to the health centre because of distance”.* (Woman, Mukonchi)

## Discussion

‘Born before arrival’ assumes that the place for childbirth should be in health facilities in accordance with the health policy. The concept is an expression of the policy and strong discourse on facility birth which partly springs out of the need to reach MDG 4 and 5. The most striking message that emerged was that users trusted the safety, confidentiality and privacy in health facilities at the time of childbirth. This worked as motivation to give birth in health facility. However, experiences related to responsiveness of health care providers, cost of buying gloves and antiseptics and fear of delivering on the way discouraged women from seeking birth care at health facilities. Cultural perceptions that related prolonged labour to infidelity and community values of endurance in labour promoted the use of herbal medicines and a delay in seeking professional assistance for childbirth.

The government set up strategies such as the Safe Motherhood Action campaign to increase demand for facility childbirth. The Safe Motherhood Action involved connecting communities to health facilities by utilizing TBAs, chiefs and headmen to encourage communities to have facility childbirth; providing bicycle ambulances to health centres; and building mother’s shelters at facilities for women who lived far to wait before delivery. Supply side strategies included training more midwives with direct entry programme, and provision of equipment for district hospitals to handle emergencies. However, there were still inadequate numbers of skilled health care providers in most health centres. Increasing demand with inadequate skilled health providers is likely to result in overcrowded facilities, longer waiting times, poor attitudes from overburdened providers in addition to serious shortages of supplies. Unmet expectations of the community from inadequate services are likely to threaten trust in the health system. In addition, with the shift in policy to facility childbirth, TBAs seem to be made redundant and risk losing their reputation and the communities’ trust in assisting at childbirth in remote areas. In a context like Zambia where 47% deliveries are provided by skilled providers, 30% assisted by relatives or no one, and 23% by TBAs [6], there is a risk of more women continuing to have childbirth at home without skilled assistance.

Users’ perspective of responsiveness of health providers included respect, quick attention to women in labour, flow of information from providers, and maintaining privacy and confidentiality. Responsiveness of health care providers is likely to affect future utilization by influencing trust and decision to seek care. Trust reflects a commitment to an on-going relationship [19], and is important in healthcare because it is a setting which is characterized by uncertainty and an element of risk regarding the competence and intentions of the health provider [20]. Respect for privacy and confidentiality recognizes the moral worth or dignity of patients as persons, and is essential for securing the benefits of a therapeutic relationship between the care-provider and those seeking care [21]. Some studies in sub-Saharan Africa have indicated that lack of responsiveness affects health facility utilization. In Ghana, low facility utilization was attributed to physical abuse, verbal abuse, neglect and discrimination by healthcare providers [22]. Provider attitude had a large influence on decisions to have facility childbirth in Tanzania and Ethiopia [23, 24]. In Malawi women perceived respect, privacy and confidentiality as important aspects of care, although, they did not seem to be critical about the treatment component [25]. Such values seem to be regarded highly in user-provider relationships as also seen in HIV counselling and testing services [26]. Apart from sub-Saharan Africa, a study in Bolivia reported poor responsiveness from health providers as contributing to low utilization of facilities at childbirth despite programmes to achieve universal maternal-child health services [27].

Incurred costs of buying delivery kits discourage utilization of health facilities at birth as has been seen in other parts of sub-Saharan Africa [28, 29]. Although user fees were abolished for delivery services in 2006 in Zambia, the poor socio-economic position of families placed them at a disadvantage due to costs of purchasing required items and transport. In order to reduce transport costs, mothers’ shelters were built to provide space for expectant women to wait a few weeks before delivery, but families still incurred indirect costs as women were taken away from productivity within their homes. However, in our previous survey in the area, perceived direct costs at the facility was not associated with low utilization since delivery care is free of charge [17].

Distance to the nearest health facility has been shown to be an important barrier to seeking healthcare both in terms of being an obstacle to reaching a health facility, and as a disincentive to seeking care [9]. In this study distance was a concern for many individuals and this was also revealed in our survey conducted in Kapiri Mposhi on factors associated with facility deliveries [17]. Many women tend to wait until the labour becomes prolonged or delivery difficult before deciding to seek care due to long walking distances to facilities. They experience fear to deliver on the way to the facility and this leads to experiences of shame and social embarrassment. Another study done in Zambia found that more than 70% of the urban population lived within 15 km of EmOC services, whereas in rural areas less than 30% lived within 15 km of an EmOC facility [30]. In Tanzania, a study showed decreased proportion of skilled attendance at birth with increasing distance [31].

The cultural belief that infidelity was related to obstructed labour could contribute to delay in seeking facility childbirth because when caesarean section was recommended by the health care provider, it would ‘expose infidelity’. This belief of infidelity and obstructed labour has been noted in other Zambian studies [32, 33] and elsewhere in Africa [29, 34]. Furthermore, culture expected women to show strength and endurance during labour. This was revealed by women delaying to inform their husbands at onset of labour. In Uganda, it was found that pregnancy was a test of endurance and those who delivered without signs of fear were respected [34]. In our study, drinking traditional herbs was commonly practiced and believed to prevent a difficult labour by accelerating labour and widening the pelvis. The use of traditional herbs in pregnancy and delivery has been documented in other African countries and could be toxic to both the mother and the foetus [35]. Health care providers discourage women from using herbs in pregnancy because of hazardous side effects and the denial of taking traditional herbs may deter women from use of health facilities [22]. However, educating them about the hazardous effects may alter their perceptions. Further research is needed in the Zambian setting to identify the commonly used traditional herbs in pregnancy and childbirth and their side effects as has been done in the South African setting [35].

Traditionally, in some parts of Zambia, women after childbirth are greeted by saying “mwapusukeni”. This means “you have survived” and reflects the risks that pregnant women undergo at childbirth. The policy promotion activities are intended to provide beneficial outcomes to pregnant women and new-borns. However, strong geographical and socioeconomic inequities in access to skilled birth attendance [17] reflect structural barriers which cannot be addressed by promotional activities only. The use of skilled birth attendance and emergency obstetric care is multifaceted and the influence of TBAs to promote facility health care seeking may be limited [36].

Social desirability bias appears to play a role in the community’s positive views of health facilities for childbirth. The policy on BBA makes it difficult for the community to say that they prefer home delivery. Promotional activities using TBAs and community leaders, whom they respect, seem to influence responses on inquiry about preference for place of childbirth. However, in this qualitative study we were able to obtain information on aspects pertaining to issues that discourage individuals and communities from facility childbirth. Possible limitations in this study were that some interviews were conducted at health facilities. Additionally, two of the interviewers for the IDIs have health background. These could have influenced responses from participants by showing willingness to utilize health facilities since most women appeared to be aware of the health policy requirement of facility childbirth. However, the participants in the FGDs had the advantage of group dynamics and freely discussed their concerns about health services and responsiveness of health providers. Research assistants were used for part of data collection in the IDIs, but they were experienced in conducting in-depth interviews and having an assistant with a background other than health was an added advantage for participants to freely express their views. The tape-recorded information helped to countercheck the transcribed data as this could be understood by the first author. Therefore, the limitations did not greatly affect the reliability of data. The use of different data collection techniques of FGDs and IDIs, and different categories of participants and from different areas increased the validity of the results. External validity is limited to the study setting of Kapiri Mposhi community.

## Conclusion

Trust and perceived quality of care were important and influenced health care seeking at childbirth. Safety, privacy and confidentiality within health facilities encouraged the community for facility childbirth. However, accessibility to facilities with skilled birth attendance, responsiveness of health providers, costs incurred due to non-availability of supplies and cultural values surrounding endurance at childbirth discouraged care seeking at birth. Interventions that include both the demand side and the supply side of services and prioritizing needs of the community could help improve trust and utilization of facilities at childbirth and help accelerate efforts to achieve MDG5.

## Electronic supplementary material

Additional file 1: **Topic guide used in the focus group discussions (REACT project).** (PDF 98 KB)

Additional file 2: **Interview guide used in the in-depth interviews in Kapiri Mposhi Zambia (REACT project).** (PDF 166 KB)
