# Supplementary material for: ‘Born before arrival’: user and provider perspectives on health facility childbirths in Kapiri Mposhi district, Zambia
Source: BMC Pregnancy Childbirth. 2014 Sep 16;14:323. doi: 10.1186/1471-2393-14-323 (PMC4171557; doi:10.1186/1471-2393-14-323)
Supplement: Supplementary file 1 — Additional file 1: Topic guide used in the focus group discussions (REACT project). (PDF 98 KB) [file 12884_2014_1187_MOESM1_ESM.pdf]

Topic guide for focus group discussions (FGDs)

Guidelines WP6-9

Setting: FGDs among these 4 groups:
Female outpatients (x 2)
Male outpatients (x 2)
Pregnant women attending ANC (x 2)
Adolescents/youth aged 18-24 (x 2)

SOPs have been developed. The following are guidelines on the themes to be discussed - with focus on equity, quality and trust - and how it relates to the selected programmes (HIV, generalised care, malaria, and obstetric care)

Questions developed for use in the population-based survey are seen as indicator questions for studying changes over time relevant to equity, quality and trust by programme. The research team members should therefore make sure that they are familiar with this questionnaire. The ideal setting will be that many of the same themes included in the indicator questions can also be brought up at the groups level and possibly more penetrating discussion.

Outpatients (men and women separately)

General care
Introduction: We would like to learn about your perceptions of the health services. We would particularly like to know if the health services at the health facilities you know are trusted among people, and if they are perceived to be accessible and of good quality.

* How are the official health services in this area? OR What is your experiences with…
* What health service / health facility /-ies do you trust more / less?
  - Dispensary, health centre, hospital, local healing (government-voluntary agency?)
  - Probe: why? Examples? (such as , good relationship with provider, information, respect, privacy, previous experience)
  - Probe: (1) "With which kind of illnesses do you go to which services (traditional healer, public services, private providers)? (2) "Why: drug availability, previous experience, cost, attitude of provider, peer/social pressure, trust?"
  - What should change for you to trust the dispensary / health centre / hospital more?
* What is your experience regarding the availability of drugs / medicine?
  - At the dispensary, health centre, hospital, local healers?
* What is your experience with the conduct of the health providers (courtesy, confidentiality, privacy, respect)?
  - At the dispensary, health centre, hospital, local healers?
  - Differing categories of providers? (nurse assistants, nurses, physicians, healers)
  - In what way could health providers modify their conduct to improve your confidence in them / your respect for them?

Malaria
Introduction: Malaria is a major cause of illness and death. We would like to ask about your experience with preventive and treatment options.

* What measures do people in this area take to prevent malaria infection?
* Do you have experience with mosquito nets / mosquito nets treated with insecticide (ITN)?
  - Have you heard about / seen the ITN nets?
  - How easy / difficult is it to get these nets?
  - Why are you using / not using an ITN net?
* What is the availability of drugs for malaria treatment?
  - At the dispensary, health centre, hospital, local healing?

HIV
Introduction: Many people are also infected with HIV. We would like to ask about the preventive and treatment options in this area.

* If someone would like to know his / her HIV status, where would he / she go for a test?
  - How easy / difficult would it be to get a test?
* What are your experiences with HIV prevention programs?
  - Which campaign or group have presented the most trust-worthy solutions? (peer education, community health workers, media, particular health facilities)
  - To what extent are condoms useful to prevent HIV infection?
  - Are condoms easily available in this area?
  - What are people's perceptions about condoms?
* How are people who are infected with HIV treated?
  - By the community?
  - By health providers (dispensary, health centre, hospital, healers)?
* How easy is it for someone infected with HIV to get access to treatment?
  - Perceptions about the treatment options?

Thank you very much for your time!

Adolescents/youth aged 18-24

Introduction: We would like to learn about young people's perceptions of the health services. We would particularly like to know if the health services at the health facilities you know are trusted among young people, and if they are perceived to be accessible and of good quality.

General care

* How are the official health services in this area? OR What is your experiences with…

* What health service / health facility /-ies do you trust more / less?

- Dispensary, health centre, hospital, local healing (government-voluntary agency?)
- Probe: why? examples?
- Probe: to what extent does trust in different health services differ with what people suffer from? (with 'diffuse / chronic illness to healers, malaria to the formal health system')
- What should change for you to trust the dispensary / health centre / hospital more?

* What is your experience regarding the availability of drugs / medicine?

- At the dispensary, health centre, hospital, local healers?

* What is your experience with the conduct of the health providers (courtesy, confidentiality, privacy)?

- At the dispensary, health centre, hospital, local healers?
- Differences between categories of providers? (nurse assistants, nurses, physicians, healers)
- In what way could health providers modify their conduct to improve your confidence in them / your respect for them?

HIV/STD

Introduction: Many people are also infected with HIV today. We would like to ask about your thoughts about the available preventive and treatment options.

* If someone would like to know his / her HIV status, where would he / she go for a test?

- How easy / difficult would it be easy to get a test?
- What are the reasons for young people not using VCT services?
- How could VCT services be modified to make them more acceptable for young people?

* What are your experiences with HIV prevention programs?

- Which campaign or group presented the most trust-worthy solutions? (peer education, community health workers, media, particular health facilities)
- Visibility of HIV programmes (map programmes observed by the participants, such as peer education, community health workers, condom campaigns, magazines, billboards etc.)
- What kinds of the involvement do the young have in HIV campaigns?
- To what extent are condoms useful to prevent HIV infection?
- Are condoms easily available in this area?
- Are condoms used by young people in this area?

* How are people who are infected with HIV treated by the community and by the health providers (at the dispensary, health centre, hospital, local healers)?

- If a family member became HIV infected, would it be better to keep it a secret?

* How easy is it for someone infected with HIV to get access to treatment?

- Perceptions about the treatment options?

* What can a person who suspects that he / she has been infected with a sexually transmitted infection do?

- Where do most people go for treatment of STI's (dispensary, health centre, hospital, local healers?)
- Availability of drugs for treating STI's?
- Perceptions of STI treatment services?

Thank you very much for your time!

Pregnant women attending ANC

General care
Introduction: We would like to learn about your perceptions of the health services. We would particularly like to know if the health services at the health facilities you know are trusted among people, and if they are perceived to be accessible and of good quality.

* How are the official health services in this area? OR What is your experiences with…
* What health service / health facility /-ies do you trust more / less?
    - Dispensary, health centre, hospital, local healing (government-voluntary agency?)
    - Probe: why? examples?
    - Probe: to what extent does trust in different health services differ with what people suffer from? (with ‘diffuse/chronic illness to healers, malaria to the formal health system..’)
    - What should change for you to trust the dispensary / health centre / hospital more?
* What is your experience regarding the availability of drugs / medicine?
    - At the dispensary, health centre, hospital, local healers?
* What is your experience with the conduct of the health providers (courtesy, confidentiality, privacy)?
    - At the dispensary, health centre, hospital, local healers?
    - Differences between categories of providers? (nurse assistants, nurses, physicians, healers)
    - In what way could health providers modify their conduct to improve your confidence in them / your respect for them?

Obstetrics
* Where do women commonly deliver in this area?
    - Why? (such as social pressure, low accessibility –distance -, high cost, low quality, low trust, previous experience)
    - What are your perceptions of deliveries at hospital/clinic level?
    - What alternatives are there?

Malaria
Introduction: Malaria is a major cause of illness and death. We would like to ask about your experience with preventive and treatment options.

* What measures do people in this area take to prevent malaria infection?
* Do you have experience with mosquito nets / mosquito nets treated with insecticide (ITN)?
    - Have you heard about them, seen them?
    - How easy / difficult is it to get them?
    - Why are you using / not using a net?
* What is the availability of drugs for malaria treatment?
    - At the dispensary, health centre, hospital, local healing?

HIV
Introduction: Many people are also infected with HIV. We would like to ask about your experience with preventive and treatment options.

* If someone would like to know his/her HIV status, where would he/she go for a test?
    - How easy / difficult would it be easy to get a test?
* What did you learn at the antenatal clinic about mother to child transmission of HIV?
    - Knowledge about possibilities of avoiding HIV transmission from mother to child?
    - What did the midwife inform about infant feeding options for HIV positive women?
* What are your experiences with HIV prevention programs?
    - Which campaign or group present the most trust-worthy solutions? (peer education, community health workers, media, particular health facilities)
    - To what extent are condoms useful to prevent HIV infection?

- Are condoms easily available in this area?

* How are people who are infected with HIV treated by the community and by health providers (dispensary, health centre, hospital)?
  - If a family member became HIV infected, will it be better to keep it a secret?
* How easy is it for someone infected with HIV to get access to treatment?
  - Perceptions about the treatment options?

Thank you very much for your time!
