# Supplementary material for: ‘Born before arrival’: user and provider perspectives on health facility childbirths in Kapiri Mposhi district, Zambia
Source: BMC Pregnancy Childbirth. 2014 Sep 16;14:323. doi: 10.1186/1471-2393-14-323 (PMC4171557; doi:10.1186/1471-2393-14-323)
Supplement: Supplementary file 2 — Additional file 2: Interview guide used in the in-depth interviews in Kapiri Mposhi Zambia (REACT project). (PDF 166 KB) [file 12884_2014_1187_MOESM2_ESM.pdf]

In-depth interview guide for maternal mortality

Section 1: socio-demographic characteristics

- Age, marital status, educational level, occupation
- Number of years in marriage, number of pregnancies, number of live children
- Length of stay in the area

Section 2: Managing a pregnancy

- What does pregnancy mean to you?
- What diseases are suffered by women during pregnancy in this area?
- Of the diseases you have stated, which one is most common among pregnant women in this area? Why?
- When a pregnant woman in this area suffers from one of the diseases you have mentioned during pregnancy, where do they go for diagnosis and treatment? Why? Probe
- Have you ever suffered from any disease during any one of your pregnancies? How did you diagnose and treat it? Why?
- What foods and medicines are taken by pregnant women during pregnancy in this area? Why? When? What?
- Who else is responsible for managing the pregnancy and taking care of the pregnant woman?
- Have there been any incidences in this area where a woman a) died with the pregnancy? B) died during delivery? C) died within 42 days (6 weeks) after giving birth?
- What caused the death?
- If you take the last 10 years, and from your own observations, has the number of women dying with pregnancy, during pregnancy, or 42 days after delivery increased, decreased or remained the same? Explain you answer. Probe
- From your own observations when did you see maternal mortality highest in this area? Why? Which circumstances contributed to that?
- What do you think about HIV testing for pregnant women? Do think people are forced to have the test?

Section 3: Labour and delivery

- Where do most women prefer to deliver their pregnancies in this area? Why?
- Where do you as a woman prefer to deliver your pregnancies? Why?
- On average how long does your labour take? Probe – hours or days?
- Who is the first person you inform when labour starts? Why?
- As labour progresses, is it monitored? How? By who? With what?
- What is usually the environment in which you deliver? Pregnant women

- What is usually the environment within which delivery occurs? TBAs
- Who is around during delivery? Why?
- How far is the nearest health facility? What facilities and human resource is there conducive for your personal satisfaction during delivery? Probe for explanation, why who, what?
- How far is the nearest TBA? What facilities are there conducive for your personal satisfaction during delivery? Probe for explanations, why, who what?
- From your observations, what are some of the reasons behind some women preferring to deliver at home? Why, what, who?

Section 4: management of a woman after delivery

- Are there any activities after delivery a woman undertakes or are done on her by other people? Who, what activities, why?
- Are there any types of foods, medicines recommended for a woman to take after delivery? What and why?
- What are the sources of these foods and medicines?
- Are there activities a woman after delivery is not allowed to be involved in? What, why, who has disallowed this?
- How long is a woman supposed to observe this? Probe in terms of days, weeks, months.
- Have you ever observed these after delivering any of your pregnancies? Why, how long, what activities? How beneficial was observing this to your health?
- When you look at the facility and the home environment, which one do you believe/think meets your desired privacy criteria, confidentiality, autonomy, dignity, social support, comfort? Probe for explanations for each criteria.

Comments

Besides what we have discussed, would you suggest anything else that would help reduce maternal mortality and generally improve the health of pregnant women in a) this area b) the country

Thank you very much for your time.
